# A novel opioid/pramipexole combination treatment for the management of acute pain: a pilot study

**DOI:** 10.3389/fpain.2024.1422298

**Published:** 2024-09-24

**Authors:** Cara Girardi, Joseph Duronio, Ryan Patton, Kevin O’Brien, Stefan Clemens, Kori L. Brewer

**Affiliations:** ^1^ Brody School of Medicine at East Carolina University, Greenville, NC, United States; ^2^ Department of Biostatistics, School of Public Health at East Carolina University, Greenville, NC, United States; ^3^ Department of Physiology, Brody School of Medicine at East Carolina University, Greenville, NC, United States; ^4^ Department of Emergency Medicine, Brody School of Medicine at East Carolina University, Greenville, NC, United States

**Keywords:** morphine, dopamine, D3 agonist, adjuvant, rats, opioid

## Abstract

**Purpose:**

Despite their dangerous side effects, opioid drugs remain a standard of care for moderate to severe pain with few alternatives. Strategies to maintain the analgesic effects of opioids while minimizing the associated risks are needed. Pre-clinical studies have shown using a dopamine 3 receptor (D3R) agonist as an adjuvant to morphine provides superior analgesia against painful stimuli compared to morphine alone. Our objective was to test if adjunct treatment with a D3R agonist can lead to a reduction in opioid use while maintaining effective analgesia.

**Patients and methods:**

This study was set up as a double-blinded, placebo-controlled randomized trial. Enrollment included acute renal colic patients presenting to the emergency department, from which patients were randomized to either the “control” or “study arm”. The control group received standard treatment of care (morphine, 0.1 mg/kg; i.v.) and an oral placebo pill. The experimental group received half-dosed morphine and oral pramipexole pill (0.25 mg). Pain measurements including a numerical pain scale and visual analog scale were collected from enrollees at baseline and every subsequent 15 min.

**Results:**

A total of 19 patients completed the study, 10 in the experimental arm and 9 in the control arm. During the study period, effective analgesia (50% decrease from baseline) was achieved in 80% of patients in the experimental arm vs. 33.3% in the control arm.

**Conclusion:**

Our pilot clinical trial demonstrated that D3R recruitment can serve as an effective adjuvant to low-dose morphine for control of renal colic pain and potentially other acute pain conditions.

**Clinical Trial Registration:**

ClinicalTrials.gov, identifier, (NCT04160520).

## Introduction

Opioid analgesics are among the most prescribed class of medications in the US. While opioids may be essential for controlling pain and other sensory disorders under acute conditions, the rates of misuse/abuse and accidental overdose involving prescription opioids has continuously increased since 1999, leading to the ongoing opioid epidemic (1). Clinicians have been challenged to find alternatives to opioid analgesics and current medical guidelines call for minimizing opioid doses in those cases where opioids are required for both acute and chronic pain (1–3). At the same time, the decision not to use opioids for certain conditions may lead to undertreatment of pain and reduced quality of life for those patients. Therefore, new regimens for pain that are highly effective but come with fewer risks are an urgent need for patients and physicians.

Pre-clinical studies have shown that using dopamine 2/3-like receptor agonists as an adjuvant to morphine provides superior analgesia against painful stimuli compared to morphine alone (4–6). This effect is maintained even when the dose of morphine is lowered to a dose that does not provide analgesia on its own (6). Pramipexole, a drug commonly used to treat Parkinson's disease (2, 7–9) and Restless Legs Syndrome (8, 10–12) is a dopamine 3 receptor (D3R) -preferring agonist that has also shown efficacy in fibromyalgia (13) and in enhancing morphine analgesia (6, 14–19). A role for the D3R but not the D2R in modulating pain-related spinal cord reflexes was previously identified in a functional D3R knockout mouse model, in which D2R remained unaltered over background controls (20), and in which application of D2R agonists was unable to rescue the behavioral readout of the D3KO animal (21). More recently, the D3R partial agonist (VK4–40) significantly decreased peak oxycodone self-administration in a nonhuman primate model of opioid use disorders (OUD) (22) and the highly selective and efficacious D3R partial agonist (S)-ABS01-113 demonstrated promising translational potential for the treatment of OUD (23). Together, these data suggest that the use of D3R-preferring agonist, such as pramipexole, as an adjuvant to morphine may allow for meaningful analgesia with minimal patient exposure to an opioid drug, reducing the risks associated with that exposure. To date, no data regarding the analgesic effect of this combination of FDA-approved drugs in humans has been described.

Using renal colic as a clinical acute condition that often presents with hallmark flank pain, we sought to compare the analgesic effect of a standard 0.1 mg/kg intravenous dose of morphine vs. a half-dose of intravenous morphine (0.05 mg/kg) in combination with oral pramipexole. This population was chosen as renal colic is often severe and frequently treated with morphine (24). The primary objective was to determine if combination therapy with a reduced level of the opioid plus pramipexole provided a similar amount of analgesia as the standard the opioid therapy alone (non-inferiority study).

## Material and methods

**Figure 1:**
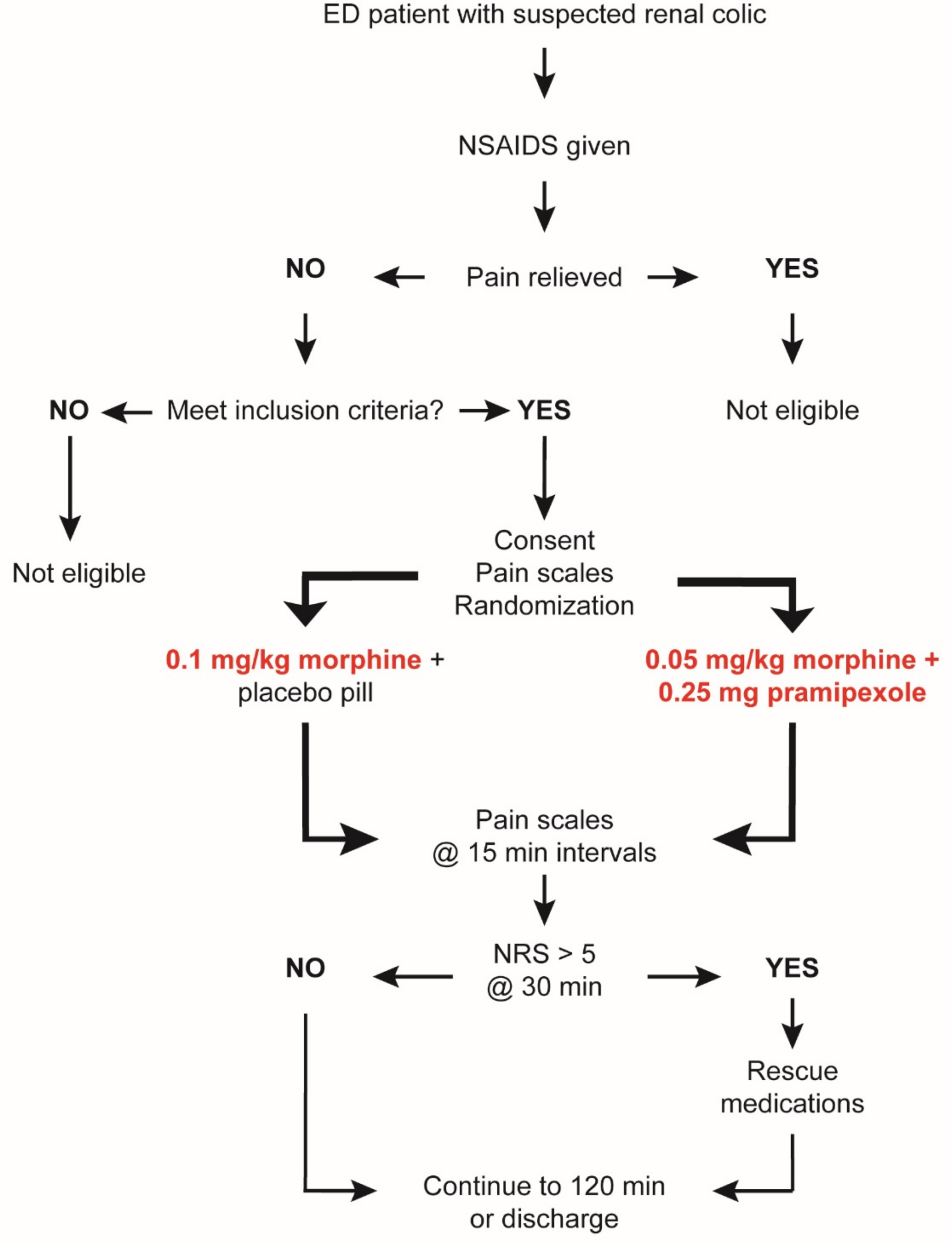
Study flow chart.

All study procedures were reviewed and approved by the University and Institutional Review Board of East Carolina University. This trial is registered on ClinicalTrials.gov (NCT04160520). A summary of the study protocol is shown in Figure 1.

### Study design

This study was a double-blinded, placebo-controlled randomized controlled trial in an academic emergency department (ED) at a rural Level I trauma center.

### Enrollment

Patients between the ages of 19 and 65 years presenting to the ED with suspected renal colic were eligible for screening. A convenience sample of patients was screened based on the inclusion and exclusion criteria described in Table 1. Patients were eligible only if they failed to obtain adequate pain relief from non-narcotic analgesic given at least 30 min prior to enrollment. Patients determined by both the research and clinical team to be eligible to participate were approached for written consent and enrolled if consent was provided. Enrollment took place from October 2019 through December 2022, with an 18-month interruption from April 2020 to January 2022 due to COVID-19 restrictions.

**Table 1:** Patient inclusion/exclusion criteria.

Inclusion criteria	Exclusion criteria
Age 19–65 years	Age <19 or > 65
ED presentation with complaint related to suspicion of renal colic	Allergy to any study medication
Failure to achieve pain relief with non-narcotic treatment prior to start of study	Known chronic renal disease
	Received IV Lidocaine during current ED visit
	^a^Currently taking any dopamine receptor agonists
	^a^Currently taking any medication with known serious contraindications to study drugs
	Unable to consent for any reason

^a^ Medications leading to exclusion from enrollment: Bromocriptine (Parlodel®). Pramipexole ER (Mirapex®). Ropinirole (Requip®). Rotigotine patch (Neupro®). Ropinirole XL (Requip XL®). Levodopa (Inbrija®, Dopar®, Larodopa®). Carbidopa/levodopa (Lodosyn®). Apomorphine (Apokyn®). Metoclopramide (Reglan®). Sulpiride (Dogmatil®) or any of the dopamine blocking antipsychotics. Cimetidine.

### Randomization

Enrolled participants were randomized into 2 arms at a 1:1 ratio using a SAS-based computer-generated randomization algorithm. The result of the randomization was blinded to the investigators, research team, and patient by having an integer (1–60) assigned to sealed envelopes that contained a card denoting if the randomization result was “control” or “study arm”. Envelopes with study assignment were secured in a locked ED pyxis that was only accessible to treating clinical team. The randomization result was only visible to the clinical team member responsible for ordering the appropriate medication and dosing. Study investigators were not in the room at the time of drug administration.

### Study arms

Eligible patients were randomized to one of two study arms: (1) Control group: received a bolus of 0.1 mg/kg intravenous morphine and an oral placebo pill. (2) Experimental group: received a bolus of 0.05 mg/kg intravenous morphine and an active pramipexole pill (0.25 mg). This lower dose of morphine is not expected to be effective for acute pain in adults on its own based on the FDA dosing guidelines for morphine (0.1–0.2 mg/kg) (25) and studies that have demonstrated the mean effective dose across all sexes and ages for post-operative pain is 0.17 ± 0.1 mg/kg (26). The investigational drug or placebo was dispensed by the investigation site pharmacy.

### Assessments

To assess analgesic effects of the 2 treatments, the following measurements were recorded: 1–10 numerical pain scale (NRS) anchored with “no pain” and “worst possible pain,” and a visual analog scale (VAS) anchored with “no pain” and “pain as bad as it could possibly be.” NRS was measured at baseline prior to administration of study drugs. VAS was not measured at baseline as the IRB would not allow for 2 pain scales to be administered prior to treatment due to the potential for delay in care. NRS and VAS were both measured every 15 min after drug treatment until patient was discharged or for 2 h (whichever came first). At 30 min after drug treatment, any patient with a NRS >5 was eligible for rescue medications (outside of study medications) at the discretion of the treating clinical team. Research team members were not involved in the decision to provide rescue medication. All patients continued in the study until the endpoint regardless of the use of rescue medications. Patients were monitored continuously for the development of any adverse event related or unrelated to study medications.

### Primary outcome measures

(1) The proportion of patients achieving effective analgesia defined as a 50% or greater improvement in pain score on the 100 mm VAS, or the NRS within 120 min of treatment in experimental vs. control groups (27). This endpoint is designed to surpass the 30% reduction in pain suggested to be the minimally important change to imply effective analgesia (28). (2) The need for rescue medications at 30 min post-treatment.

An overview of the study design is shown in Figure 1.

### Sample size calculation

Power analysis for non-inferiority trial of binary outcome (50% improvement in pain score, Yes vs. No) was performed using the following conditions:

Percent success of treatment in control group = 100%; Percent success of treatment in experimental group = 80%; Non-inferiority limit = 10; Alpha = 0.05; Power = 90%.

If there is a true difference in favor of the experimental group of 20%, then 32 patients are required to be 90% sure that the upper limit of a one-sided 95% confidence interval will exclude a difference in favor of the standard group of more than 10%.

To account for overestimation of effect in experimental group, the planned target number to enroll was set at 30/group (*n* = 60 total enrollment).

### Statistical analysis

Per protocol, the primary outcome was proportion of patients achieving at least a 50% improvement in pain score on the 100 mm VAS, and the NRS within 120 min of treatment in study vs. control groups. Initially designed as a non-inferiority trial, this approach was abandoned due to recruitment issues. A 2 × 2 contingency table (Fisher's Exact test) analyzed treatment group by effective analgesia response (Yes, No). Time to first effective analgesia response was computed as elapsed from start of treatment and analyzed using a logrank Mantel-Cox test and plotted using the Kaplan-Meier method. Due to missing data points over time, change in NRS and VAS pain scores over time was analyzed using a mixed linear model to detect the effect of treatment group on pain scores over time with slope estimates reported for each group as a measure of change over time. All statistical analysis was performed using SPSS v.27 (IBM®) and graphs created in GraphPad Prism (v.10.2.3, Dotmatics).

## Results

Twenty-one patients were enrolled in the study. 2 enrollees were withdrawn due to protocol deviations, leaving 19 patients who were randomized, 10 to the experimental arm and 9 to the control arm. Groups were similar with respect to age, gender, and race (Table 2). All patients received oral ibuprofen (800 mg) for pain control prior to enrollment and subsequent administration of study drugs.

**Table 2:** Demographics.

	Control	Experimental
Age (years) mea*n* ± SEM (Range)	43.2 ± 4.3 (42.0)	41.0 ± 2.7 (27.0)
Male		
*N* (%)	4 (46)	6 (60)
Race		
White	4	6
Black	3	2
Hispanic	1	2
Other	1	0

### Analgesia outcomes

**Figure 2:**
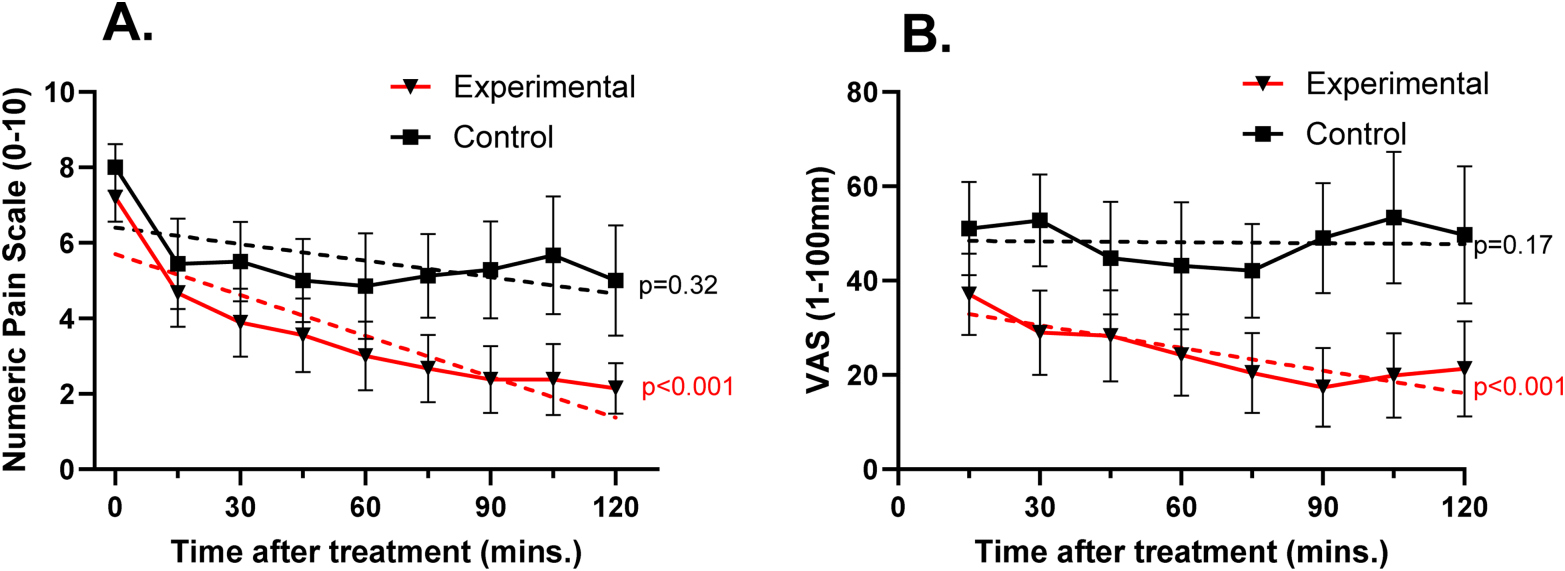
Mean (± SEM) pain ratings over time using the 0–10 numeric pain scale (**A**) and the 0–100 mm visual analog scale (**B**) dotted lines represent predicted slope based on a mixed linear model of change in score within each group over time. On the NRS, groups had similar pain ratings prior to treatment and showed a similar decrease at 15 min after treatment. Beyond 15 min, NRS and VAS pain ratings for patients in the experimental group continued to decrease while those in the control group remained constant. *p*-values indicate significance of predicted slope.

Pain ratings over time for each group are shown in Figure 2. When measured using the NRS, change in pain over time in the experimental group showed a significant slope estimate (−0.461; *p* < 0.001) while the control group did not (−0.080; *p* = 0.32). The same was seen when pain was measured on the VAS, with the experimental group showing a slope estimate of −2.84 (*p* < 0.001) and a slope estimate of −1.10 in the control group (*p* = 0.169). Not all patients provided a pain scale at all time points. This mainly resulted from patients being out of the emergency department for imaging or other treatment during a data collection time. Two patients in the experimental group and 2 patients in the control group were discharged prior to study completion. Therefore, the 90, 105 and 120-minute assessments included 8 patients in the experimental group and 7 in the control group.

The primary outcome measure of reaching effective analgesia by 120 min was achieved in 80% (*n* = 8) of patients in the experimental arm vs. 33.3% (*n* = 3) in the control arm (*p* = 0.07). No patients required rescue medications at the 30-minute time point. One patient in the control group received rescue medication (2 mg intravenous morphine) at 60 min after study drug administration and remained in the study to the 120-minute time point.

**Figure 3:**
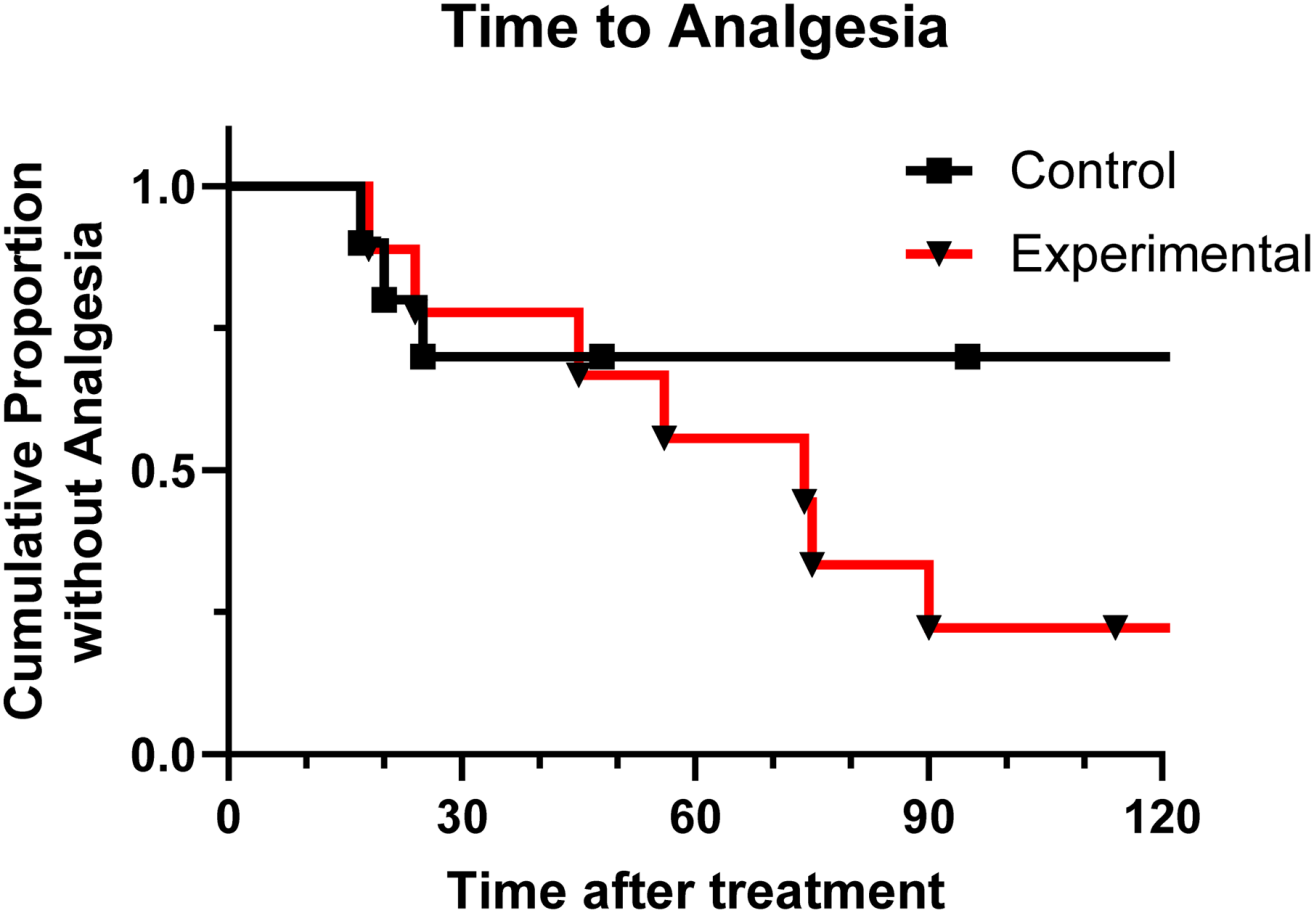
Kaplan-Meier analysis shows proportion of patients in each group that have not achieved effective analgesia over time.

Figure 3 shows the percent who had not achieved effective analgesic response at each time point. Descriptive values of the predicted mean and median time to first effective analgesic response, had there been no time cut off, is shown in Table 3. The curves were not significantly different (*p* = 0.180). However, while at the 15-minute measurement the two arms are similar, overall more subjects in the experimental group reached first effective analgesic response than in the control group.

**Table 3:** Estimated mean and median time to first effective analgesic response.

Estimates	Control (mins.)	Experimental (mins.)	Overall (mins.)	*p*-value log-rank: mantel-cox
Mean ± SEM (95% CI)	135.7 ± 33.5 (70.1–201.3)	73.9 ± 14.5 (45.4–102.1)	111.6 ± 22.0 (68.4–154.8)	0.18
Median ± SEM (95% CI)	136.0 ± 85.0 (0.00–302.6)	75.0 ± 23.7 (28.6–121.4)	90.0 ± 39.1 (13.3–166.7)	

### Adverse events

No adverse events occurred in either group.

## Discussion

In the first clinical trial of this combination of 2 FDA-approved and commonly used drugs, we demonstrated that the D3R agonist pramipexole may be an effective adjuvant to morphine for acute pain control, demonstrated here in renal colic patients. These results support the growing literature on the ability of D3Rs to enhance opioid-based analgesia (4–6, 29, 30). This combination of morphine and pramipexole represents a potential novel therapeutic intervention for pain conditions as it reduces patient exposure to opioids and lessens the risks associated with standard opioid dosing.

The importance of these results lies in the potential to reduce the doses of opioids needed to control pain. The CDC’s 2022 Clinical Practice Guideline for Prescribing Opioids for Pain emphasizes the need for physicians to reduce the risks associated with opioid pain therapy while maintaining flexible, patient-centered care. Specifically, the risk of overdose is known to be opioid dose dependent (31), and studies have described the primary factor associated with long-term use in opioid-naïve patients as being higher initial prescribed doses (32, 33). The CDC Guideline specifically states that when opioids are initiated in opioid-naïve patients with pain, “clinicians should prescribe the lowest effective dosage” and that the benefit of pain relief should outweigh the risk in all patients prescribed opioids (3). While there is evidence to show that non-opioid therapies can be as effective as opioids for many types of acute pain (7, 34–36), opioids will remain important for treating severe acute pain related to traumatic injury, postoperative pain, burns or other conditions for which alternative pain treatments (i.e., NSAIDS) are contraindicated or ineffective. In these cases, the addition of pramipexole as an adjuvant treatment may provide a means for clinicians to harness the analgesia that opioids provide while still meeting the goal of minimizing the doses needed to provide effective pain relief.

There is considerable overlap between dopaminergic and mu-opioid receptors (MORs) expression and pathways in brain and spinal cord (37, 38), and we have shown in preclinical studies that the use of dopamine D3Rs in combination with an opioid to treat pain improved analgesia without the typical side effects of opioids. Specifically, we demonstrated that the D3R agonist pramipexole enhanced morphine's analgesic effects in animal models of acute and chronic pain conditions (6). Importantly, we could produce effective analgesia in this neuropathic pain model with doses of morphine and pramipexole that on their own were ineffective. This is mirrored in the current study in which we were able to achieve analgesia in patients with renal colic using half of a commonly used dose of morphine.

The current literature fails to fully explain how D3R modulation impacts opioid-induced analgesia, but we demonstrated earlier that functional D3Rs are necessary to achieve morphine analgesia (39, 40). D3Rs and mu-opioid receptors (MORs) can form functional heterodimers in the ventral horn of the spinal cord (41), and they are co-localized in the dorsal horn of the spinal cord (17), a site critical for modulating nociceptive input. D2 receptors are also co-expressed with MORs in the same neurons in the striatum (38) where extensive interactions between the dopamine and opioid systems have been demonstrated.

While the current study used an acute pain condition in which to test this drug combination, there is pre-clinical data to suggest that dopamine modulation may also contribute to the effective management of chronic conditions such as neuropathic pain (9, 42–44) and migraine pain (44). In rodent studies, this drug combination did not lead to the development of tolerance with chronic administration (17). Additionally, on a test of reward potential (conditioned place preference), animals receiving the drug combination do not develop a preference for the drug-paired chamber while those receiving morphine alone did (6).

Our study did not include a pramipexole alone group, but neither pre-clinical or clinical data support the idea that pramipexole is a strong analgesic on its own. In rodent models, pramipexole has only shown to provide pain control at doses well above the human equivalent dose of 0.25 mg used in our study (45, 46). The ability of pramipexole to provide pain relief in humans has also been studied and, when administered chronically, was shown to provide some degree of pain relief in patients with fibromyalgia (13). However, the dose of pramipexole in that study was increased weekly to a dose 18 times higher than that used in our study, with effects on pain measured weekly. Additionally, more than 50% of the patients in this study were also taking a narcotic during treatment with pramipexole so these data may further demonstrate a synergistic effect of D3-agonists and opioids (13). The CDC recommends nonopioid therapies for subacute and chronic pain, noting that opioids should only be used if the expected benefits for pain relief outweigh the risks to the patient. With the addition of pramipexole as an adjuvant, it may be possible to maintain the benefit of potent analgesic effect of opioids over time at doses that do not risk the development of tolerance, dependence, and addiction. Clinical trials will be required to determine if this is the case.

The results of this clinical study support what has been described in animal models regarding the enhancement of morphine analgesia with the addition of pramipexole. However, several questions remain that need to be addressed prior to full translation of this research to the clinic. Just one dose combination of drugs was tested. This work must be replicated on a larger scale, optimizing the ratio of drugs used so that the doses of both morphine and pramipexole can be minimized. In addition, while pramipexole is highly effective in treating RLS in the short term, long-term exposure is often associated with the gradual emergence of augmentation (8, 11, 47–50). However, this typically occurs only with doses much higher than those used in this study, and after treatment durations that span years. Additionally, the combination of drugs needs to be tested across a wide range of painful conditions to determine if the effect is universal. Most importantly, it is critical to fully assess the addictive potential and abuse potential of this drug combination with acute and long-term use to ensure that effective doses do not put patients at the same risk as existing opioid regimens do.

The study is primarily limited by low power created by the small sample size. For the primary outcome of 50% pain reduction at 120 min, our study reached a power of 55% to detect a significant difference between treatment groups. Based on these preliminary results, post-hoc power analysis indicates that a minimum of 16 patients per group would be needed to reach 80% power with *p* < 0.05. This goes beyond our goal of showing non-inferiority of the experimental treatment would suggest that the experimental treatment is potentially superior to morphine alone for renal colic.

We also report limited data with regards to the effects of both treatments. There is a need to also collect data on short-term side effects of the treatments, including nausea, sedation, euphoria and effects on standard vital signs. It is also critical to study other types of acute and chronic pain conditions before generalizing the potential of this treatment beyond renal colic. This study does not address the issue of tolerance and dependence that might occur with long-term use of the drug combination. Additionally, other D3 agonists may have better selectivity and perform better as an adjunct to morphine. While pre-clinical trials have addressed these issues, there is no data in humans on the effects of chronic use of this combination.

## Conclusion

This small-scale pilot clinical trial demonstrated that activation of the D3R system through administration of pramipexole can enhance opioid-mediated analgesia in patients with renal colic, an acute pain condition, while at the same time reducing the overall amount of the opioid used. This novel therapeutic approach has the potential to minimize the quantity of prescribed opioids and thus reduce the risks of side adverse side effects of these drugs, including overdose, tolerance and addiction.

## Data Availability

The raw data supporting the conclusions of this article will be made available by the authors, without undue reservation.
